# Acute Infrarenal Abdominal Aortic and Bilateral Common Iliac Artery Occlusions in an Elderly Female: A Case Report

**DOI:** 10.7759/cureus.59913

**Published:** 2024-05-08

**Authors:** Qambar Hasan, Chandler M Griffin, Alexander Doerffler

**Affiliations:** 1 Osteopathic Medicine, Nova Southeastern University Dr. Kiran C. Patel College of Osteopathic Medicine, Fort Lauderdale, USA; 2 Graduate Medical Education, Broward Health North, Deerfield Beach, USA; 3 Emergency Medicine, Broward Health North, Deerfield Beach, USA

**Keywords:** vascular interventional radiology-evar, acute aortic occlusion, embolectomy, infrarenal abdominal aorta, abdominal ct angiography

## Abstract

Acute aortic occlusions (AAOs) are rare vascular emergencies associated with high morbidity and mortality. Presenting signs and symptoms vary but typically involve the lower extremities and include mottled skin with diminished pedal pulses, paresis, and severe pain. Prompt recognition and imaging are necessary to prevent rapid deterioration, which can lead to loss of limb or death. Treatment includes surgical or endovascular interventions based on patient-associated risk factors and clot location.

We present a 76-year-old female who arrived at the emergency department with an AAO involving the infrarenal abdominal aorta and bilateral common iliac arteries. Efficient physical examination and utilization of computed tomography with angiography of the abdomen and pelvis allowed for the appropriate recognition of the AAO and subsequent successful surgical embolectomy. This case report underscores the importance of an expeditious clinical and radiographic evaluation in patients presenting with lower extremity pain and weakness.

## Introduction

Acute aortic occlusions (AAOs) are rare vascular events with an incidence of 3.8 per one million person-years, a statistic derived from the identification of cases in a nationwide vascular database maintained for the Swedish population [1]. AAO typically presents with signs and symptoms of acute lower extremity ischemia, which include pain, paresis, mottled extremities, and diminished pulses [2]. More proximal aortic clots can occlude the superior mesenteric artery (SMA), renal arteries, or spinal arteries and present with associated ischemic complications involving the gastrointestinal system, kidneys, and spinal cord [2]. A retrospective study reported 30-day mortality after surgical intervention for AAO of 27.7%, patients requiring hemodialysis at 21.5%, and amputation at 15.4% [3]. These life-threatening complications make a prompt and accurate diagnosis crucial. Without a thorough history and physical examination, this condition may be mistaken for more common causes of lower extremity pain and weakness, such as peripheral neuropathy, acute ischemic stroke, chronic claudication, or acute lumbar disc herniation.

The treatment for AAO involves prompt surgical revascularization to the pelvis and lower extremities [3]. Various modalities exist that can be employed to surgically treat an AAO, including, but not limited to, catheter-directed thrombolysis, axillobifemoral bypass, aortobifemoral bypass, aortoiliac thromboembolectomy, and aortoiliac stenting [3]. Data comparing the various modalities are limited, and one of the most comprehensive comparative analyses took place at a single institution, encompassing AAO cases from 2006 to 2017 [3]. Axillobifemoral bypass was shown to have lesser operative morbidity as a modality and the most commonly employed treatment mortality; however, this was at the expense of durability [3,4]. The confidence in the axillobifemoral bypass modality is further strengthened by the procedure being utilized most commonly in the treatment of AAO as per a nationwide database in Sweden [1]. Aortoiliac thromboembolectomy was reserved for patients considered high-risk and with minimally diseased aortoiliac segments at this institution [3]. If limb amputation was indicated, it was typically performed prior to revascularization [3]. Revascularization was prioritized when there was potential for a viable limb [3].

A comprehensive retrospective chart review at the aforementioned institution of patients presenting with an AAO between 2006 and 2017 has shown an overall postoperative 30-day mortality of 27.7% and a mortality of 26.2% when hospitalized [3]. A vascular database maintained at a separate institution has shown that patients treated for AAO between the years 2005 and 2013 demonstrate a 30-day postprocedure mortality rate specifically for AAOs occurring distal to the renal arteries to be 18% [2]. It was also shown that an age above 60, elevated lactate levels, and motor deficits of the lower extremities on presentation were associated with a higher 30-day mortality [3]. Common complications of surgical intervention for AAO involved acute kidney injury, respiratory failure, and cardiovascular issues such as cardiac arrest, myocardial infarction, and new-onset atrial fibrillation [3]. Finally, the median length of hospital stay after intervention for AAO was shown to be 12 days [3].

## Case presentation

A 76-year-old female with a past medical history of obesity and atrial fibrillation controlled with warfarin presented to the emergency department (ED) with bilateral lower extremity weakness and pain. The patient noted that her symptoms began while making breakfast when she lost the ability to move her lower extremities.

On presentation to the ED, her vitals included an afebrile temperature of 36.5°C, sinus tachycardia with a heart rate of 130 beats per minute, respiratory rate of 18 breaths per minute, pulse oximetry of 98% in room air, and notable systolic hypertension with a widened pulse pressure at 180/83 mmHg. During the physical examination, it was observed that the patient was in severe distress, which was evident from her overall appearance. Her skin was significant for a cool, mottled appearance of the lower extremities with dusky-appearing toes. The cardiovascular examination revealed regular tachycardia without palpable femoral, popliteal, or pedal pulses and delayed capillary refill of both feet. The neurological examination was significant for flaccid lower extremities with diminished sensation. The rest of her examination was unremarkable. Due to high clinical suspicion, she was sent for a stat computed tomography (CT) angiogram of the abdomen and pelvis with a differential diagnosis inclusive of AAO and thoracic aortic dissection.

Laboratory measurements performed included a complete blood count, blood chemistry studies, and coagulation studies (Table 1).

**Table 1:** Patient laboratory values

Laboratory measurement	Patient value	Reference range
Sodium	139 mmol/L	136–146 mmol/L
Potassium	4.0 mmol/L	3.5–5.0 mmol/L
Chloride	104 mmol/L	95–105 mmol/L
Bicarbonate	17 mmol/L	22–28 mmol/L
Anion gap	18 mmol/L	4-12 mmol/L
Blood urea nitrogen	30 mg/dL	7–18 mg/dL
Creatinine	1.8 mg/dL	0.6–1.2 mg/dL
Calcium	10.1 mg/dL	8.4–10.2 mg/dL
Albumin	3.9 g/dL	3.5–5.5 g/dL
Total protein	7.8 g/dL	6.0–7.8 g/dL
Alkaline phosphatase	122 units/L	25–100 units/L
Aspartate aminotransferase	26 units/L	12–38 units/L
Alanine aminotransferase	18 units/L	10–40 units/L
Total bilirubin	0.6 mg/dL	0.1–1.0 mg/dL
Magnesium	1.8 mg/dL	1.5–2.0 mg/dL
Troponin I	0.01 ng/mL	≤0.04 ng/mL
White blood cell count	11.0 thousand/mcL	4.5–11.0 thousand/mcL
Hemoglobin	14 g/dL	Female: 12.0–16.0 g/dL
Hematocrit	41%	Female: 36%–46%
Platelet count	204 thousand/mcL	150–400 thousand/mcL
Prothrombin time	20.2 seconds	11–15 seconds
International normalized ratio	1.7	≤1.1 (2.0-3.0 effective therapeutic range on warfarin)
Partial thromboplastin time	31.1 seconds	25–40 seconds
Serum lactate	3.4 mmol/L	0.7–2.1 mmol/L

**Figure 1:**
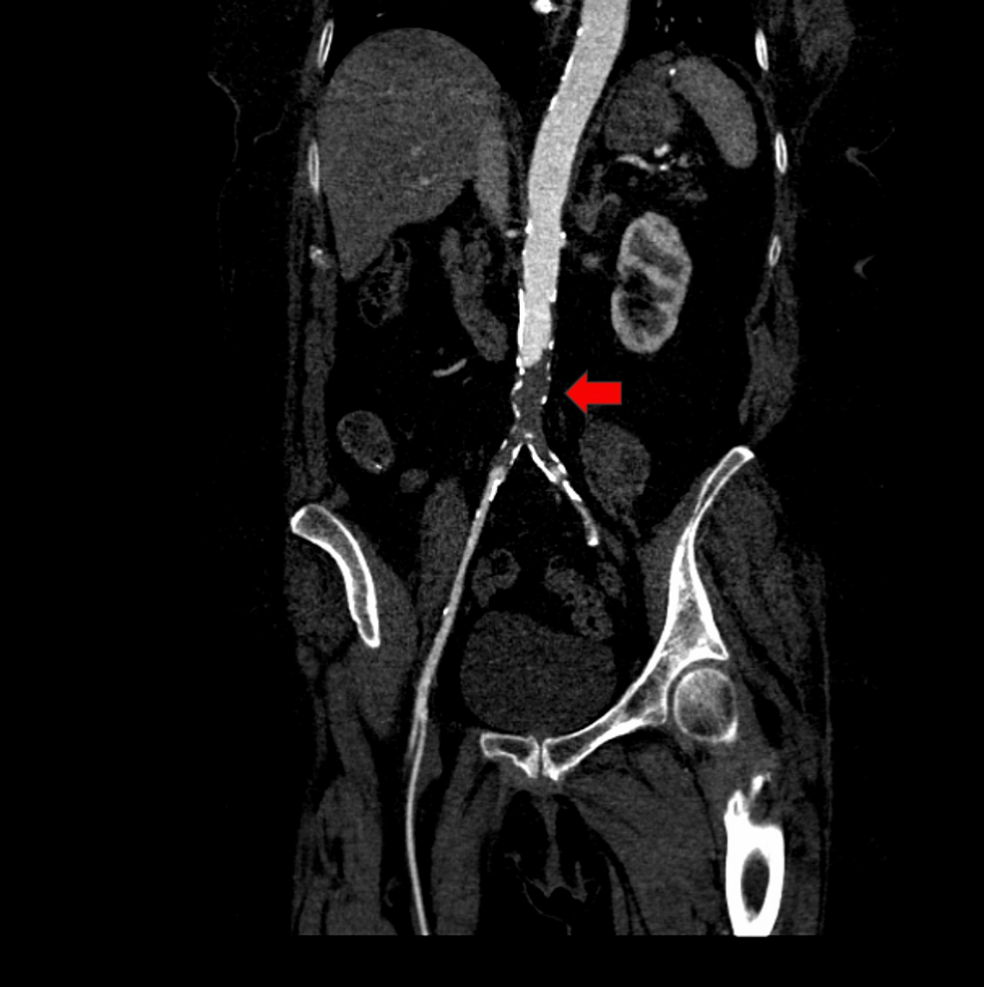
Computed tomography with angiography of the abdomen and pelvis showing occlusion of the infrarenal abdominal aorta and right and left common iliac arteries (red arrow)

CT angiogram of the aorta demonstrated occlusion of the infrarenal abdominal aorta along with occlusions of the right and left common iliac arteries with notable reconstitution of the right and left external iliac arteries secondary to collaterals (Figure 1). The celiac artery was also found to be occluded at its origin. Additionally, 50% stenosis was noted in the SMA due to plaque formation, and mild stenosis was observed in the inferior mesenteric artery secondary to calcified plaque. Plaque formation was also observed at the origins of both the right and left renal arteries without significant stenosis.

The results of the CT angiogram warranted surgical intervention involving embolectomies of the abdominal aorta, bilateral iliac arteries, and bilateral femoral arteries. Her vascular surgery service estimated an ischemia time of four hours (time of symptom onset until presentation in the operating room). Following embolectomies, the operative note demonstrated strong femoral and pedal pulses. On postoperative day 1, her mild acute renal injury and lactate acidosis resolved. Ultimately, the patient did not require amputation and, on postoperative day 6, was subsequently discharged home with home-based physical therapy. At a six-week follow-up visit with her cardiologist, they noted a normal gait and pedal pulses. They recommended continuing all home medications and following up with a pulmonologist regarding her insomnia.

## Discussion

Primary mechanisms typically implicated in the development of an AAO involve embolism and thrombosis [5]. Risk factors for embolism, as in this case, include the female gender and the presence of heart disease (e.g., atrial fibrillation), whereas risk factors for thrombosis involve smoking and diabetes mellitus [5]. Furthermore, the elderly female patient in this case was noted to be obese, a condition that has been associated with hypercoagulability and can potentially lead to deviations in aortic blood flow [6]. Future research efforts should address the potential link between obesity and AAO because obesity is a known risk factor for other more common causes of intra-abdominal vascular catastrophe, such as aortic dissection and aneurysms [7,8].

Nevertheless, the increased use and availability of CT angiography reflect a paradigm shift from traditional angiography to diagnose AAO formally [9]. In fact, CT angiography is now considered the first-line imaging modality for the rapid diagnosis of primary aortic occlusions. This term refers to AAO without aortic atherosclerosis or aneurysm and further describes our case [9]. CT angiography also offers the potential to rapidly rule out or identify unexpected causes of AAO that could have ramifications on the treatment plan and outcome for the patient [3].

Additionally, the surgical modality of embolectomy chosen in this case differs from the most common procedure typically chosen to treat AAO, the axillobifemoral bypass [3]. This procedure was chosen after ensuring the patient was an appropriate candidate, as patients with chronic atherosclerosis involving the affected area or prior iliac stents are deemed not eligible for this option [3].

## Conclusions

This case report presents an AAO in a high-risk patient based on age, along with the presence of obesity and atrial fibrillation. Due to high clinical suspicion and prompt diagnostic testing, our patient received the most appropriate surgical treatment, allowing her to make a complete recovery and avoid dire complications such as amputation. Additionally, the patient was able to be discharged home in a timeframe that was significantly less than the median length of stay. Because AAO is very rare, a future meta-analysis and systematic review may be the only way to provide definitive diagnostic and treatment recommendations for this life-threatening condition.
